# Functional relationship of *AtABCG21* and *AtABCG22* in stomatal regulation

**DOI:** 10.1038/s41598-017-12643-6

**Published:** 2017-10-02

**Authors:** Takashi Kuromori, Eriko Sugimoto, Haruka Ohiraki, Kazuko Yamaguchi-Shinozaki, Kazuo Shinozaki

**Affiliations:** 1 RIKEN Center for Sustainable Resource Science, Wako, Saitama, 351-0198 Japan; 2 Graduate School of Agricultural and Life Sciences, The University of Tokyo, Bunkyo-ku, Tokyo, 113-8657 Japan

## Abstract

Stomatal regulation is important for water transpiration from plants. Stomatal opening and closing are controlled by many transporter proteins in guard cells. AtABCG22 is a member of the ATP-binding cassette (ABC) transporters and is a stomatal regulator; however, the function of AtABCG22 has not yet been determined fully, although a mutant phenotype included a significant effect on stomatal status. Here, we further investigated the function of the *AtABCG22* gene and its functional relationships with other subfamily genes. Among close family members, we found a functional relationship of stomatal phenotypes with *AtABCG21*, which is also expressed specifically in guard cells. Based on an analysis of double mutants, adding the *atabcg21* mutation to *atabcg22* mutant partially suppressed the open-stomata phenotype of *atabcg22*. Multiple-mutant analyses indicated that this suppression was independent of abscisic acid signaling in guard cells. We also found that *atabcg22* mutant showed a unique time course-dependent phenotype, being defective in maintenance of stomatal status after initial stomatal opening elicited by light signaling. The function of AtABCG22 and its relationship with AtABCG21 in stomatal regulation are considered.

## Introduction

Stomata consist of a pair of guard cells, a unique cell type with an important role in regulating water transpiration from plant surfaces. Many regulators in guard cells have been shown to function in the closing or opening of stomata. For example, *open stomata1* (*ost1*)/*snf1-related protein kinases 2e* (*srk2e*) mutants defective in abscisic acid (ABA) signaling have been isolated as major open-stomata (OST) mutant due to the inability to close its stomata^1,2^. Additionally, cell membrane transporter proteins are important as stomatal regulators^3,4^, because guard cells are isolated symplastically from neighboring cells^5–7^.

The superfamily of ATP-binding cassette (ABC) transporters is one of the most abundant protein families in nature. These proteins are broadly conserved from prokaryotes to higher eukaryotes in all phyla that use energy to transport substrates in an ATP-dependent manner against concentration gradients. Plant genomes, in particular, have large ABC families of more than 100 genes, indicating that some ABC transporters likely have important roles in plant-specific developmental and environmental responses^8,9^.

In plant ABC subfamilies, the ABCG subfamily is the largest, composed of both ‘half-size’ and ‘full-size’ transporters. In *Arabidopsis*, 28 gene members have been classified as AtABCG half-size transporters^8^, about half of which have been described in the literature. For example, AtABCG1 and AtABCG16 are required for pollen nexine layer formation, and AtABCG2, AtABCG6, and AtABCG20 are required for suberin barriers^10–12^. AtABCG9 is involved in pollen coat maturation, related to steryl glycosides, in concert with a full-size transporter, AtABCG31^13^. AtABCG11, AtABCG12, and AtABCG13 are involved in transporting precursors of wax and cutin to the epidermis^14–18^. AtABCG14 controls the root-to-shoot translocation of cytokinins and plant development^19–21^. AtABCG19 has a role in antibiotic resistance^22^. AtABCG25 is involved in ABA transport and responses^23^. AtABCG26 is essential for exine formation, by transporting polyketide sporopollenin precursors^24–29^. Most of these transporters are consequently related to roles in maintaining water content or in prohibiting water loss from plant bodies or pollen cells.

In addition to the members above, we previously isolated AtABCG22, which also functions in retaining water in plants^30^. Mutant *AtABCG22* plants show increased water transpiration and drought susceptibility, suggesting a relationship with ABA function. However, we additionally found enhanced phenotypes of *atabcg22* mutant which exhibited an additive effect to ABA signaling or ABA biosynthesis, so that the function of AtABCG22 in guard cells remains to be determined.

Here, we show that *AtABCG22* has a functional relationship with *AtABCG21*, which is a closely related but as yet unanalyzed member of the ABCG subfamily in *Arabidopsis*. We also examined an *atabcg22* mutant phenotype involved in stomatal regulation.

## Results

### *AtABCG21* gene expression patterns in plant organs

**Figure 1:**
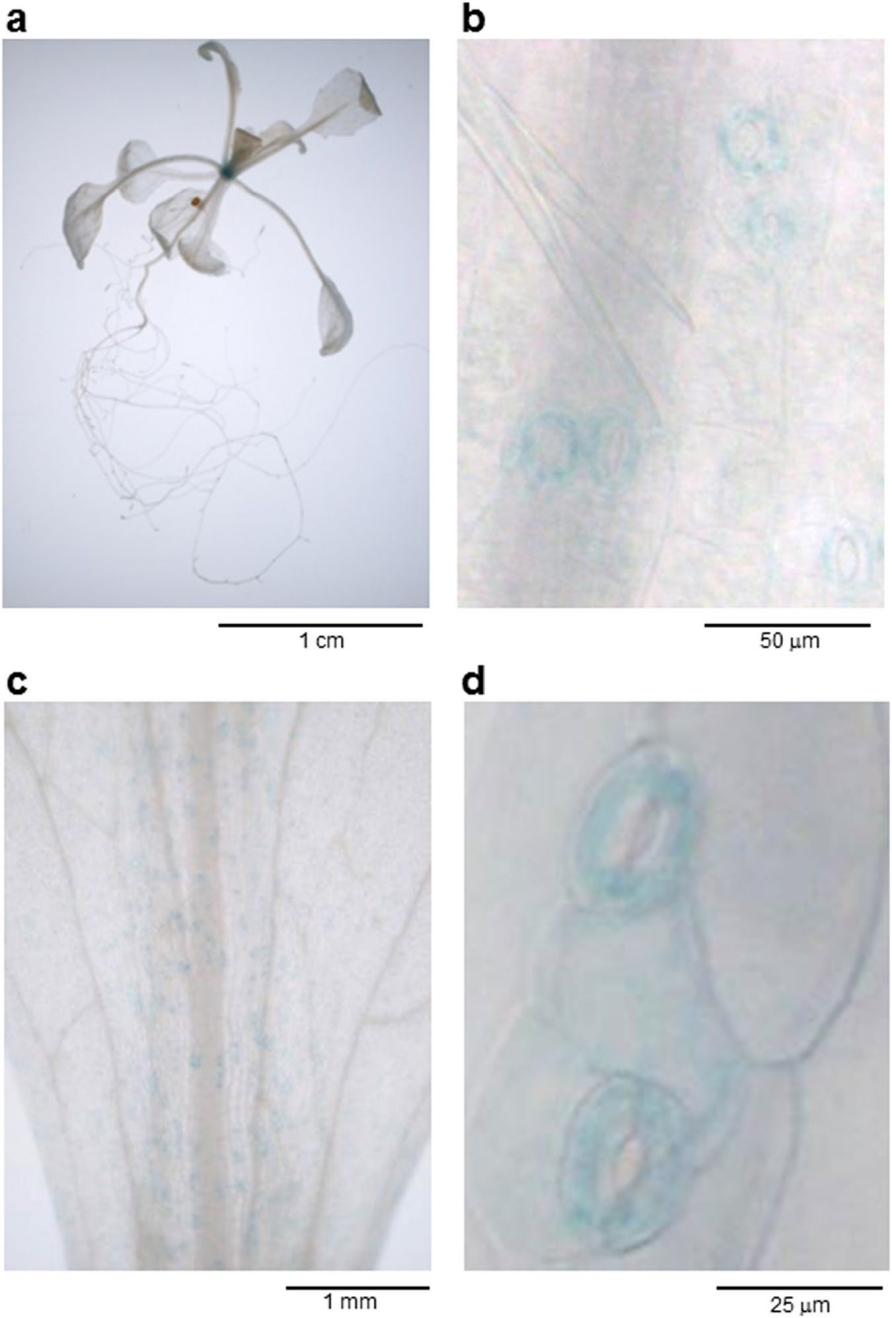
*AtABCG21* gene expression patterns based on GUS staining. (**a**) Whole seedling of a 2-week-old plant. (**b**) Magnified image of a leaf in (**a**). (**c**) Leaf of a 5-week-old plant. (**d**) Magnified image of (**c**). In both growth stages, guard cells were mostly stained.

We previously reported AtABCG22 was involved in stomatal regulation, because *atabcg22* mutants that exhibited a typical open-stomata (OST) phenotype^30^. To study the relationship between AtABCG22 and other ABCG members that were closely related based on a phylogenetic tree^17,30^, we focused one of the family members, *AtABCG21*. To investigate the gene expression patterns of *AtABCG21* in wild-type (WT) tissues, we used ~2 kb of the *AtABCG21* promoter region (*pAtABCG21*) to drive expression of a *β-glucuronidase* (*GUS*) reporter gene. In *pAtABCG21::GUS* transgenic plants, GUS activity of the transformants was detected in guard cells of seedlings, but not in roots (Fig. 1a,b). The leaf surface was also stained in adult plants, and GUS staining was detected primarily in guard cells in magnified images (Fig. 1c,d). This gene expression pattern was similar to that of *AtABCG22* ^30^, suggesting a guard-cell function for AtABCG21.

### Subcellular localization of the AtABCG21 protein

**Figure 2:**
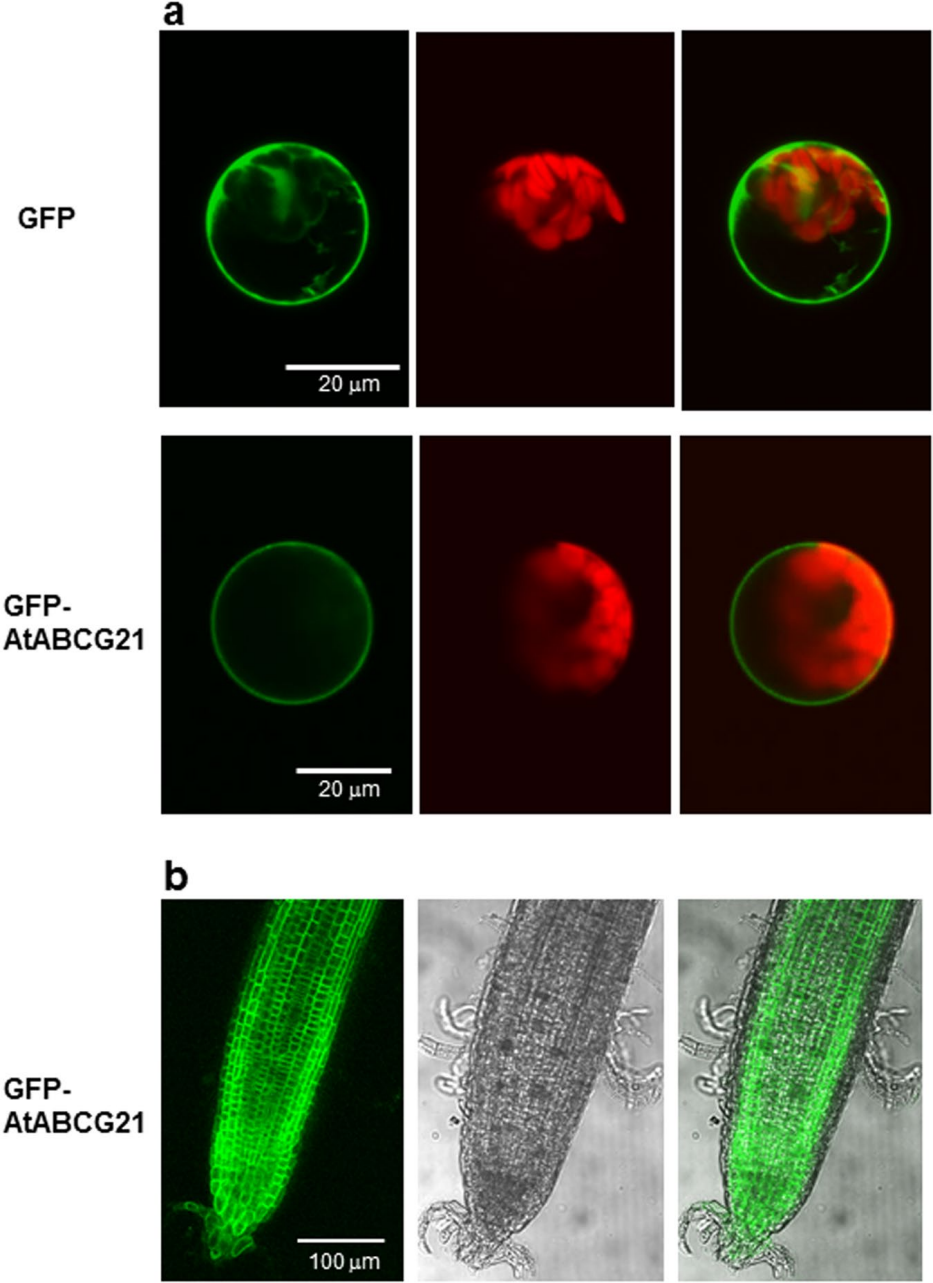
Subcellular localization of AtABCG21. (**a**) Transient expression of GFP protein and GFP-AtABCG21 fusion protein in *Arabidopsis* protoplasts. The left panels show images of GFP fluorescence, the middle panels are images of chloroplast autofluorescence, and the right panels are merged images. (**b**) Subcellular localization of GFP-AtABCG21 fusion proteins in root cells in 35Spro:GFP-AtABCG21 transgenic plants. The left panel shows an image of GFP fluorescence, the middle panel is a bright-field image, and the right panel is a merged image.

We showed previously that AtABCG22 was localized to the cell membrane in plant cells^30^. To study the subcellular localization of AtABCG21, we made a construct that expressed green fluorescent protein (GFP) fused to AtABCG21 protein under the control of the Cauliflower mosaic virus 35 S promoter. The *AtABCG21* open reading frame was placed downstream of 35 S::*GFP*. The 35 S::*GFP-AtABCG21* recombinant gene was expressed transiently in *Arabidopsis* protoplasts. Subcellular localization of the fusion protein was visualized by confocal imaging of green fluorescence in protoplast cells. The green fluorescence of the GFP-AtABCG21 recombinant protein was present around the cell surface (Fig. 2a). Additionally, we transformed the 35 S::*GFP-AtABCG21* recombinant vector into *Arabidopsis* plants. GFP-AtABCG21 recombinant protein fluorescence was observed clearly around the cell surface in root cells of the transgenic plants (Fig. 2b). These results indicated that AtABCG21 was localized to the cell membrane.

### Suppression effect of an *atabcg21* mutation on an *atabcg22* mutant phenotype

**Figure 3:**
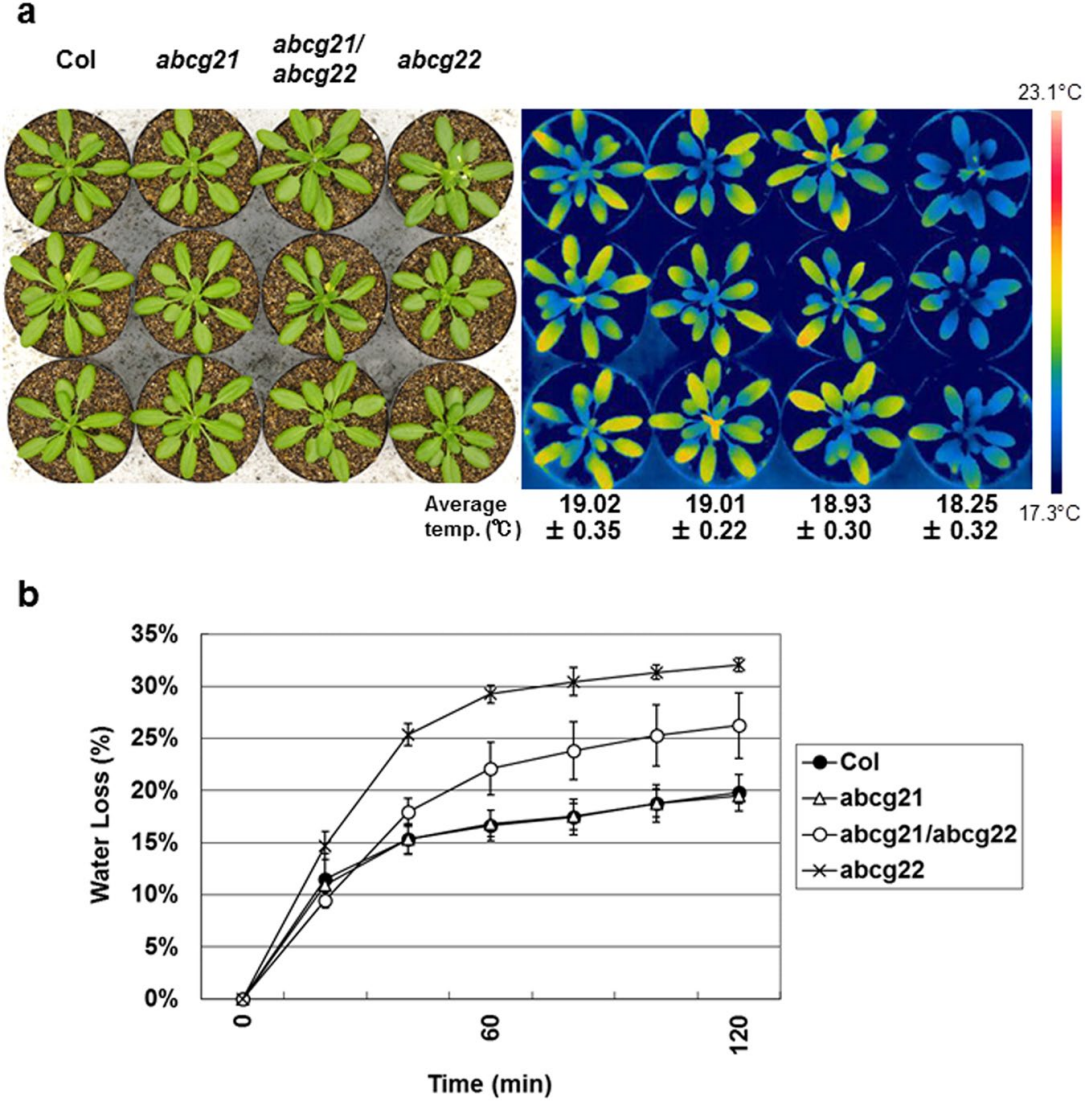
Functional relationship between *atabcg21* and *atabcg22* in transpiration phenotypes. (**a**) Thermal images of *atabcg21*, *atabcg22*, and double-mutant plants. Rosette leaves of 5-week-old wild-type plants (Col), *atabcg21* mutant plants (*abcg21*), double-mutant plants (*abcg21*/*abcg22*), and *atabcg22* mutant plants (*abcg22*) were imaged with a visible-light camera (left panel) and an infrared thermography device (right panel). Average temperatures are shown with SD under each line of thermal images. (**b**) Transpiration ratio of *atabcg21*, *atabcg22*, and double-mutant plants. Water loss in detached rosette leaves of 5-week-old plants was determined as a percentage of the initial fresh weight. Values are shown as means ± SD of the three independent plants photographed in (**a**).

We previously reported that the leaf temperature of *atabcg22* mutant plants was lower than that of WT plants under normal growth conditions, because of the OST phenotype^30^. To investigate the functional relationship between *AtABCG21* and *AtABCG22*, we isolated a homozygous T-DNA insertional mutant on *AtABCG21* by genotyping (Supplemental Fig. S1a), and confirmed this line was a gene knockout mutant (Supplemental Fig. S1b). We did not find any significant phenotype related to water transpiration for the *atabcg21* mutant plants (Supplemental Fig. S1c,d, Fig. S2a,b). However, when we crossed an *atabcg21* mutant with an *atabcg22* mutant to produce double mutants, we found that the leaf temperature of *atabcg21/atabcg22* double-mutant plants was similar to that of WT plants, indicating that transpiration was lower in the leaves of the double mutants than the *atabcg22* mutants (Fig. 3a). This was confirmed by the results of a water-loss experiment in which the rate of weight loss from detached leaves of the double-mutant plants was slower than that from detached leaves of *atabcg22* mutants, partially recovered to WT plants or *atabcg21* single mutants (Fig. 3b). These results indicated that the *atabcg21* mutation partially suppressed the OST phenotype of *atabcg22* mutants, suggesting a functional relationship between *AtABCG21* and *AtABCG22* in stomatal regulation.

### The *atabcg21* mutation does not suppress *srk2e* and *nced3* mutant phenotypes

Next, we investigated whether the *atabcg21* mutation could suppress the phenotype of other OST-type mutants, particularly those related to ABA biosynthesis or ABA signaling. We crossed *atabcg21* mutant plants with *srk2e* or *nine-cis-epoxycarotenoid dioxygenase3* (*nced3*) mutants to produce each double mutant. *SRK2E* encodes a kinase involved in cellular ABA signaling in guard cells and *NCED3* encodes a key enzyme in ABA biosynthesis^1,2,31^. Single mutants of *srk2e* or *nced3* are typical OST-type mutants, and showed lower leaf temperatures and accelerated transpiration from leaves, compared with WT plants (Supplemental Figs S1,S2). The leaf temperature and transpiration rates of the *atabcg21/srk2e* and *atabcg21/nced3* double mutants were almost the same as those of the *srk2e* and *nced3* single mutants, respectively (Supplemental Figs S1,S2). These results indicated that the *atabcg21* mutation did not generally suppress the phenotype of all OST-type mutants, but may repress *atabcg22* specifically, suggesting a specific relationship with the function of AtABCG22.

### Phenotypes of multiple-mutant plants with ABA signaling defects

**Figure 4:**
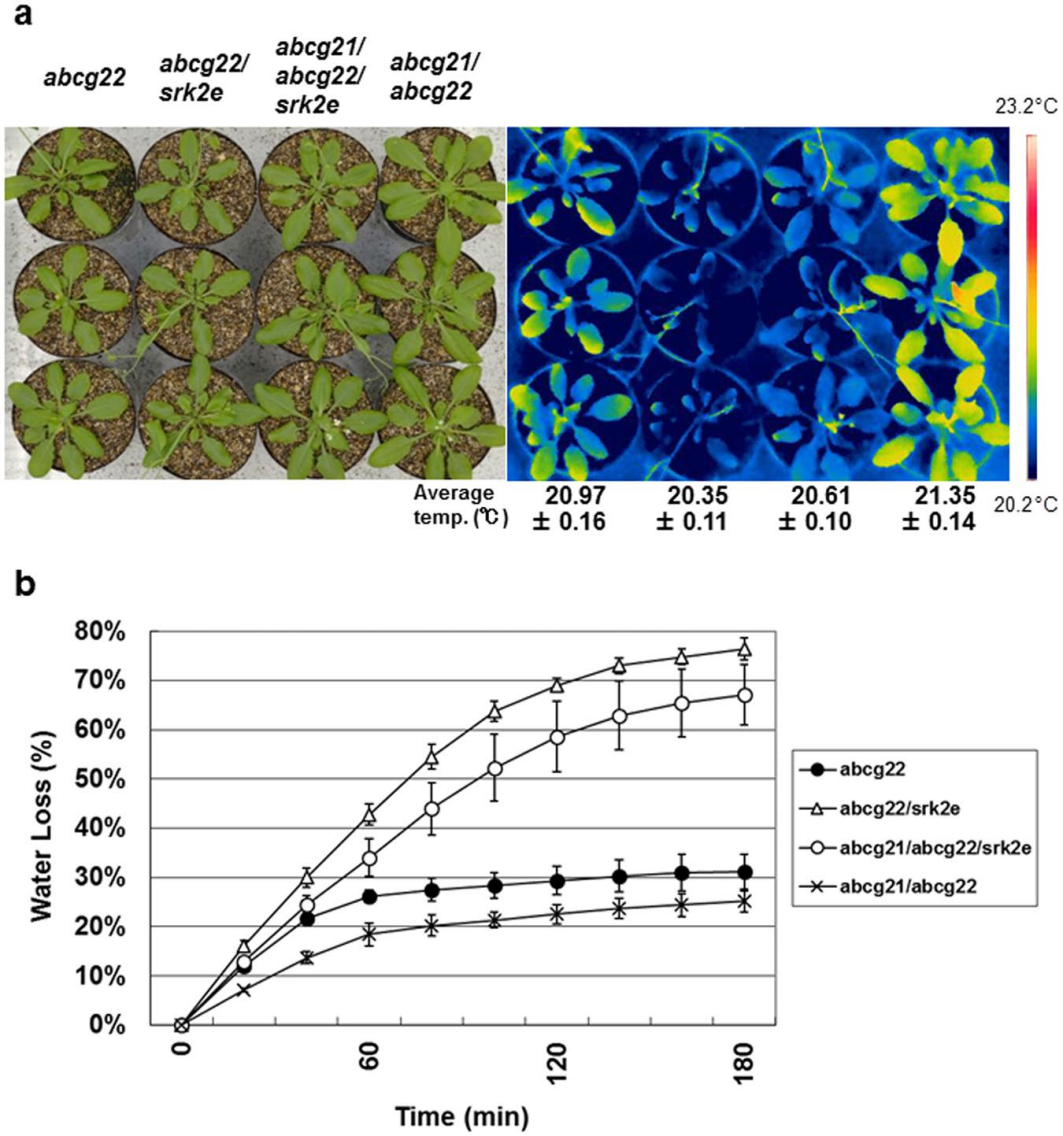
Suppression phenotype of *atabcg21* on *atabcg22* and an ABA signaling mutant. (**a**) Thermal images of double and triple mutant plants of *atabcg21*, *atabcg22*, and *srk2e*. Rosette leaves of 5-week-old *atabcg22* mutant plants (*abcg22*), *atabcg22*/*srk2e* double-mutant plants (*abcg22*/*srk2e*), *atabcg21*/*atabcg22*/*srk2e* triple*-*mutant plants (*abcg21*/*abcg22*/*srk2e*), and *atabcg21*/*atabcg22* double-mutant plants (*abcg21*/*abcg22*) were imaged with a visible-light camera (left panel) and an infrared thermography device (right panel). Average temperatures are shown with SD under each line of thermal images. (**b**) Transpiration ratio of double and triple mutant plants of *atabcg21*, *atabcg22*, and *srk2e*. Water loss in detached rosette leaves of 5-week-old plants was determined as a percentage of the initial fresh weight. Values are shown as means ± SD of the three independent plants photographed in (**a**).

When we first isolated *atabcg22* mutants, we suspected a relationship between AtABCG22 function and ABA responses. However, double mutants of *atabcg22* and ABA signaling mutants showed additive effects, so this issue was not resolved^30^. In the process of investigating whether the suppression effect of the *atabcg21* mutation on the *atabcg22* mutant was dependent on ABA in guard cells, we crossed *atabcg21/atabcg22* double-mutant plants with *srk2e* mutants to produce triple mutants. As reported previously for the enhanced phenotypes of *atabcg22/srk2e* ^30^, the leaf temperature of the triple-mutant plants, including *srk2e*, was lower than that of the *atabcg21/atabcg22* double mutants (Fig. 4a). Notably, the leaf temperature of triple-mutant plants was slightly, but significantly, higher than that of *atabcg22/srk2e* double mutants (Fig. 4a). This result was consistent with that of a water-loss experiment, in which the rate of weight loss from detached leaves of the triple-mutant plants was lower than that from detached leaves of *atabcg22/srk2e* double mutants (Fig. 4b). These results indicated that the *atabcg21* mutation still suppressed the OST phenotype of *atabcg22/srk2e* to some extent, even in a *srk2e* mutant background, having ABA-signaling defects. This suggested that the suppression effect of the *atabcg21* mutation on *atabcg22* mutants may be independent of ABA signaling in guard cells.

### Light signal-dependent phenotype of the *atabcg22* mutant

**Figure 5:**
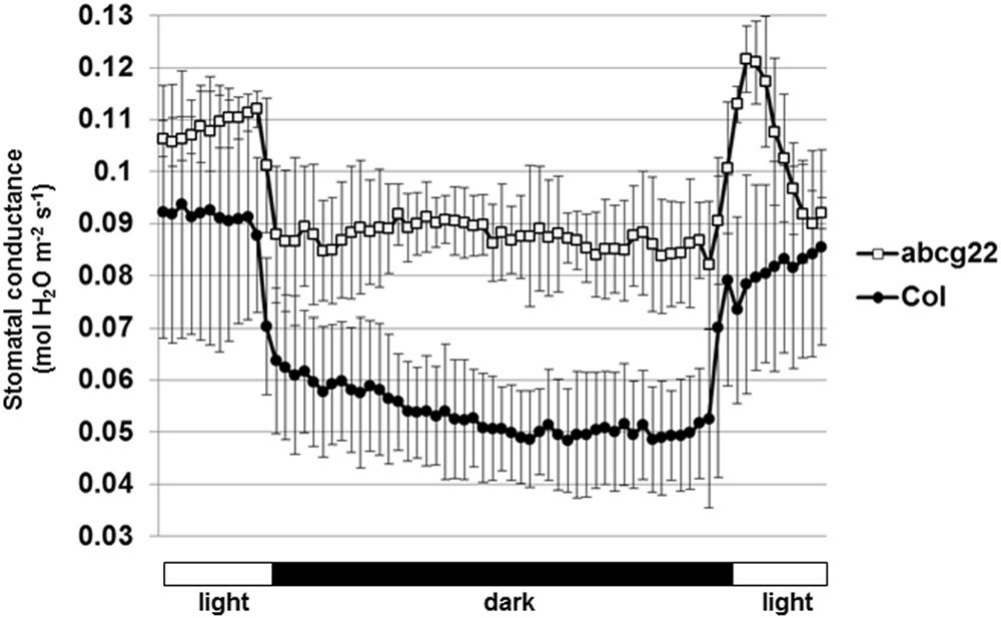
Stomatal conductance in response to changes in light status. Stomatal conductance of wild-type (Col) and *atabcg22* (abcg22) was measured using a gas-exchange system (LI-6400). Data are plotted at 10 min intervals for 12 h, including 8 h of dark, in a conditioned green house. The CO_2_ concentration of the air flow was set at 400 ppm during experiments. Values are shown as means ± SD (*n* = 3).

The *atabcg22* mutant plants exhibited lower leaf temperatures and increased water loss, indicating elevated transpiration through an effect on stomatal regulation. To address the function of AtABCG22, we measured stomatal conductance over a time course, including light and dark periods. Overall, the stomatal conductance of *atabcg22* mutants was higher than that of WT plants, consistent with having an OST-type phenotype (Fig. 5). That of *atabcg21* mutants was same as WT plants.

In this experiment, we found a unique behavior of *atabcg22* mutants in the initial period of lighting. When the light was turned on, stomatal conductance increased quickly, as in WT plants. However after this increase, it decreased rapidly in *atabcg22* mutants while it stabilized in WT plants (Fig. 5). When the light was turned off, there appeared to be a normal response in *atabcg22* mutants. These results suggest that AtABCG22 may have a function in balancing the stomatal status after light signaling for stomatal opening.

## Discussion

When we crossed *atabcg21* and *atabcg22* mutants to generate double mutants, we found that addition of the *atabcg21* mutation partially suppressed *atabcg22* mutant phenotypes (Fig. 3). In case of signal transduction, when the phenotype of the first mutation was suppressed by the addition of the second mutation, it could suggest bypassing the first mutation by the second mutation in the signaling pathway. However, the mutated genes in this case encode transporter proteins, rather than signaling factors. Furthermore, both genes are expressed predominantly in guard cells, and both proteins were shown to be localized to the cell membrane (Figs 1,2). Additionally, the result that *atabcg21* mutations do not repress other OST-type mutants may indicate a specific relationship between AtABCG21 and AtABCG22 (Supplemental Figs S1,S2).

Accordingly, the simplest interpretation would be that the two transporters have opposite activities in transporting the same substrate. Generally, it has been assumed that eukaryotic ABC proteins uniformly transport a substrate present at the side of the membrane where the nucleotide-binding domain is located to the other side of a membrane; for example, transporters localized to the cell membrane are usually efflux transporters, moving substrates from the cytosol to the outside. However, recent findings show that at least some plant ABC transporters can also act in the opposite direction, as influx transporters, moving substrates from the apoplastic space to the cytosol^9,32–34^.

ABA is a strong effector enhancing stomatal closure. Several AtABCG family members have been reported to be ABA-transporting factors^23,34,35^. *AtABCG22* transcripts are enriched by drought treatment even under mild conditions^36^. We suspected that AtABCG21 and/or AtABCG22 would be related to ABA, but genetic combination of multiple mutations did not show this (Fig. 4). Another experiment showed that the stomatal response to exogenous ABA was intact in *atabcg22* mutants, suggesting that AtABCG22 is not involved in ABA uptake into guard cells directly, although AtABCG22 plays a role in initiating stomatal closure due to reduced air humidity^37,38^. Targeted substrates of AtABCG21 and AtABCG22 are still unknown and remain to be investigated.

Light, especially blue light, causes positive signaling for stomatal opening^39^. On the other hand, a report that light-dependent stomatal movement of *ost1-2*, an allele of *srk2e*, appeared to be unaffected suggested that light-induced stomatal opening is essentially independent of ABA signaling^1^. In our time-course measurements, we found that *atabcg22* mutant plants could actually respond to light, but then showed rapid attenuation of stomatal opening (Fig. 5). When the light was turned on, cellular light-induced signaling in guard cells was triggered instantly, followed by stomatal opening^40^. Based on the time course-dependent phenotype of the *atabcg22* mutant, AtABCG22 may have a unique function in establishing stable stomatal status in the initial hour after light exposure.

## Methods

### Plant materials and observations

Plants were grown in soil under well-watered conditions at 22 ± 2 °C and 60–70% relative humidity under a 16/8-h light/dark cycle. The *atabcg21* mutant was a T-DNA-tagged mutant obtained from the Arabidopsis Biological Resource Center (SAIL_786_G09). The *atabcg22* mutant used in this study was reported previously as the *atabcg22-2* (salk_113844) allele, and the *srk2e* and *nced3* mutants have been described previously^30^.

Thermal images were captured using an infrared thermography device (T620; FLIR). Average temperatures were calculated by FLIR Tools. Water-loss experiments were performed using rosette leaves detached from 5-week-old plants, as described previously^30^.

### Visualization of expression sites by GUS staining

For *AtABCG21* promoter-driven GUS expression lines, a 2-kb AtABCG21 promoter region was amplified using KOD plus polymerase (Toyobo) with the primers AtABCG21pro_Fw (5′-CACCGACACCTAAACAAATAGACTTCGTGA-3′) and AtABCG21pro_Rv (5′-CTAGAGAAGGAAAGAGAGATAG-3′), cloned into the pENTR/D-TOPO vector (Invitrogen), and then integrated into the GUS-fusion vector, pBGGUS. The plasmid was then electroporated into *Agrobacterium tumefaciens* to generate transgenic plants by floral dipping. GUS staining and observation of GUS-stained plants were performed as described previously^30^.

### Subcellular localization of AtABCG21

Transient expression assays using mesophyll protoplasts from *Arabidopsis* were performed as described previously^41^. To build the 35 S::GFP-AtABCG21 construct, a fragment of the *AtABCG21* coding region was amplified by PCR from *Arabidopsis* cDNA with the primer set AtABCG21_Fw_EcoRV (5′-AACGATATCATGATGCCTCCTAATGAGCA-3′) and AtABCG21_Rv_NotI (5′-ATAGCGGCCGCTCACAAGTTCCTTAGAGCTA-3′). The amplified fragment was ligated between the *Eco*RV and *Not*I sites of the pGKX-NsGFP vector^42^. The same vectors were electroporated into *Agrobacterium* to generate transgenic plants.

### Gas exchange measurements

Stomatal conductance was assayed in rosette leaves of 5- to 7-week-old wild-type and *atabcg22* plants using a portable gas exchange system (LI-6400; LI-COR). The air flow was set to 200 μmol s^–1^, and the humidity of the air was not regulated, but the CO_2_ concentration of the air was controlled at 400 ppm using a CO_2_ cylinder during the experiments.

## Electronic supplementary material

Supplemental Figures
