# Supplementary material for: Functional relationship of AtABCG21 and AtABCG22 in stomatal regulation
Source: Sci Rep. 2017 Oct 2;7:12501. doi: 10.1038/s41598-017-12643-6 (PMC5624933; doi:10.1038/s41598-017-12643-6)
Supplement: Supplementary file 1 — Supplemental Figures [file 41598_2017_12643_MOESM1_ESM.pdf]

Functional relationship of *AtABCG21* and *AtABCG22* in stomatal regulation

Takashi Kuromori [1] *, Eriko Sugimoto [1], Haruka Ohiraki [2], Kazuko Yamaguchi-Shinozaki [2] & Kazuo Shinozaki [1]

Supplemental Figures

Supplemental Figure 1

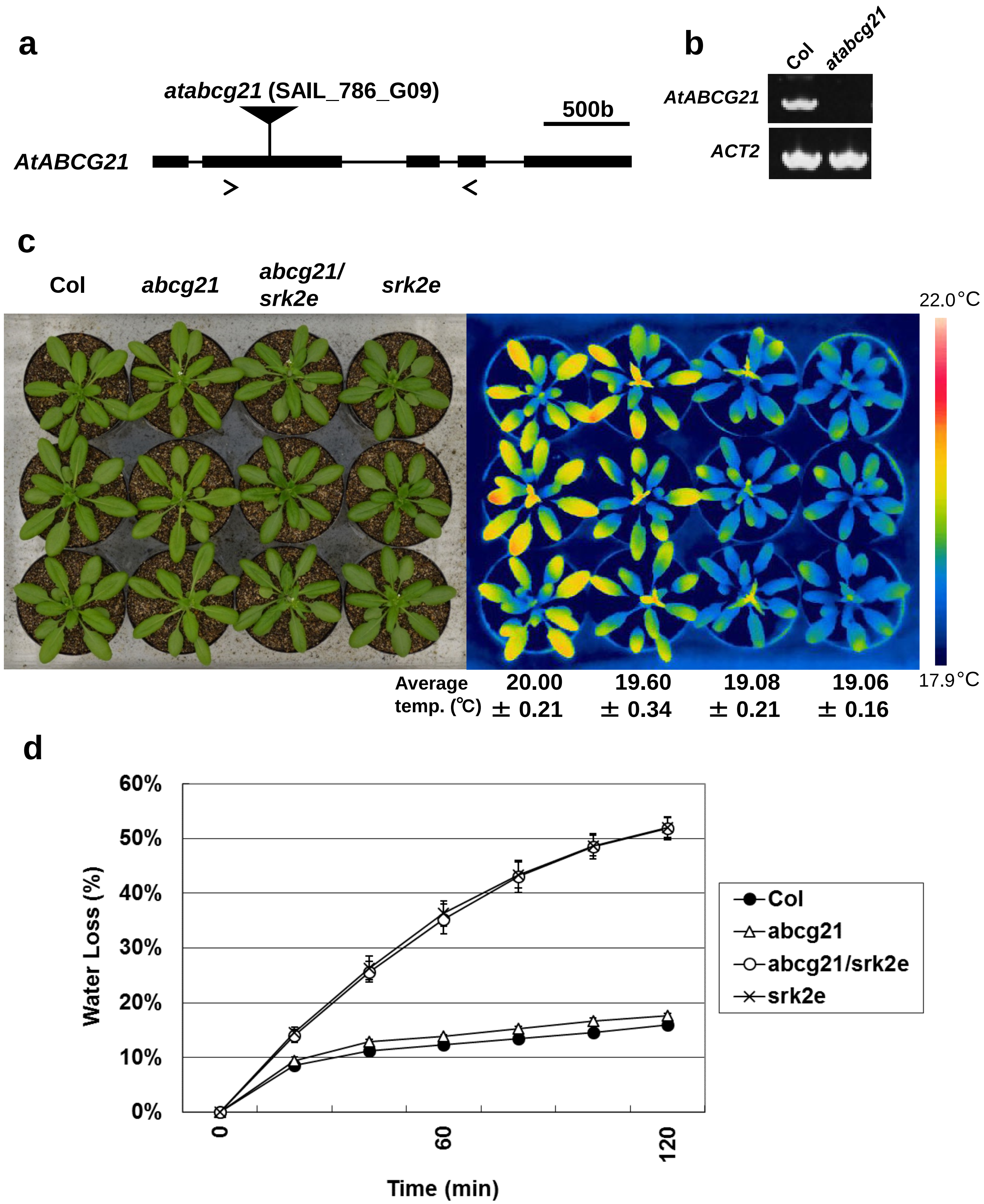

Supplemental Figure 1. **No functional relationship between *atabcg21* and *srk2e* in ABA signaling in transpiration phenotypes**.

(a) T-DNA insertion site of the *atabcg21* mutant. Square boxes represent exons and black bars represent introns. T-DNA insertions indicated by a black triangle are based on PCR genotyping with the primer set (5'-GAAACTAGAGCAGGTGGAGATG-3') and (5'-AAATCCAAAAACAACGTACGAC-3').

(b) RT-PCR analysis of *AtABCG21* transcripts in wild-type plants and in *atabcg21* mutants. Total RNAs were prepared from rosette leaves of 5-week-old wild-type plants (Col) and the *atabcg21* mutant, and checked by semi-quantitative PCR with the primer set (5'-GGTATAGTCAAACCAGGGGAGT-3') and (5'-CAAGAGGCCTGAAAGTAAAGAA-3'), indicated by arrow heads in (a). *Actin2* (*ACT2*) was used as a reference, with the primer set (5'-GACCTGCCTCATCATACTCG-3') and (5'-TTCCTCAATCTCATCTTCTTCC -3').

(c) Thermal images of *atabcg21*, *srk2e*, and double-mutant plants. Rosette leaves of 5-week-old wild-type plants (Col), *atabcg21* mutant plants (*abcg21*), double-mutant plants (*abcg21*/*srk2e*), and *srk2e* mutant plants (*srk2e*) were imaged with a visible-light camera (left panel) and an infrared thermography device (right panel). Average temperatures are shown with SD under each line of thermal images.

(d) Transpiration ratios of *atabcg21*, *srk2e*, and double-mutant plants. Water loss in detached rosette leaves of 5-week-old plants was determined as a percentage of the initial fresh weight. Values are shown as means ± SD of the three independent plants photographed in (c).

Supplemental Figure 2

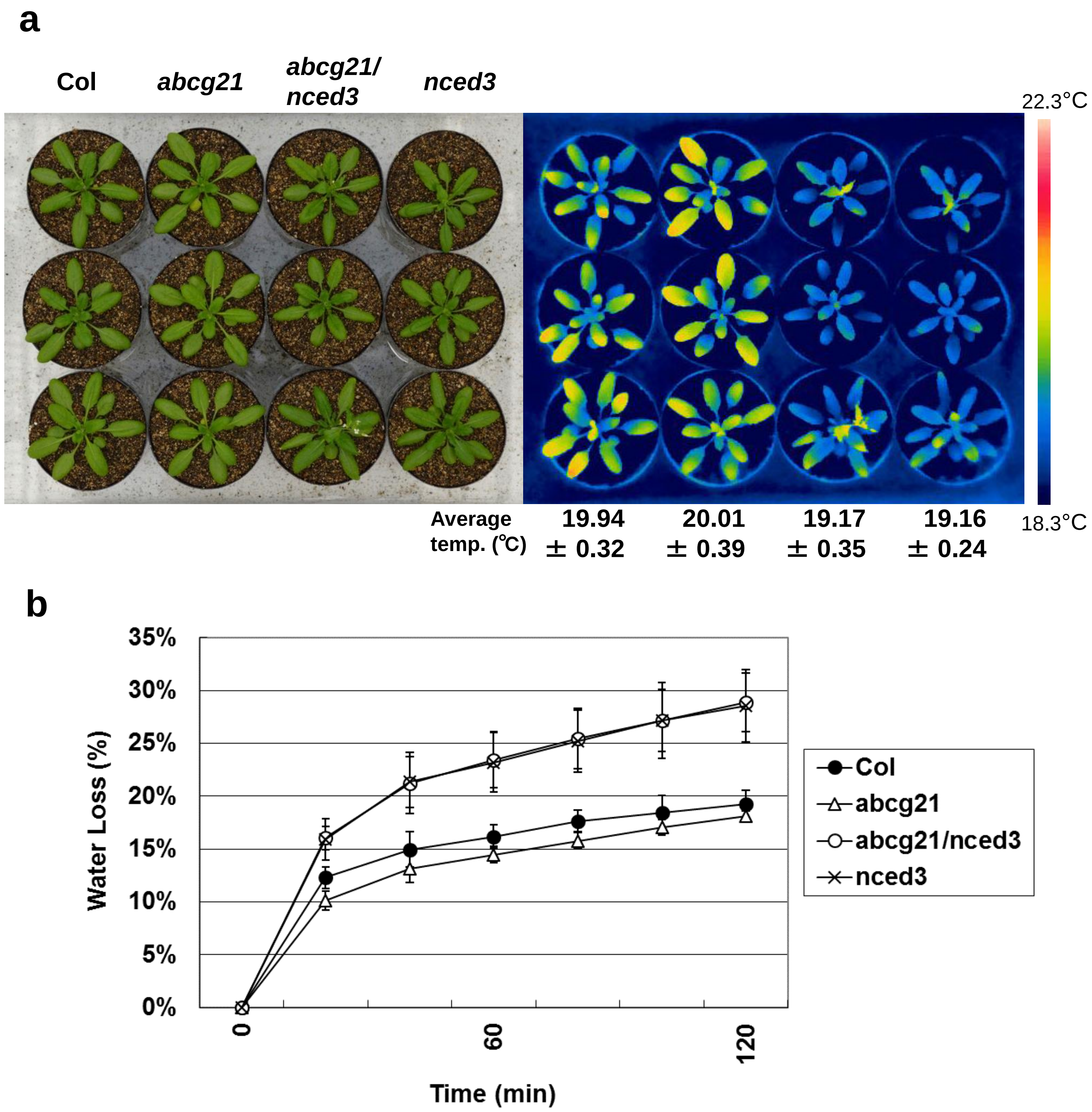

Supplemental Figure 2. No functional relationship between *atabcg21* and *nced3* in ABA biosynthesis in transpiration phenotypes.

(a) Thermal images of *atabcg21*, *nced3*, and double-mutant plants. Rosette leaves of 5-week-old wild-type plants (Col), *atabcg21* mutant plants (*abcg21*), double-mutant plants (*abcg21*/*nced3*), and *nced3* mutant plants (*nced3*) were imaged with a visible-light camera (left panel) and an infrared thermography device (right panel). Average temperatures are shown with SD under each line of thermal images.

(b) Transpiration ratios of *atabcg21*, *nced3*, and double-mutant plants. Water loss in detached rosette leaves of 5-week-old plants was determined as a percentage of the initial fresh weight. Values are shown as means ± SD of the three independent plants photographed in (a).
